# Solvent and A-Site Cation Control Preferred Crystallographic Orientation in Bromine-Based Perovskite Thin Films

**DOI:** 10.1021/acs.chemmater.3c00075

**Published:** 2023-05-25

**Authors:** Juanita Hidalgo, Yu An, Dariia Yehorova, Ruipeng Li, Joachim Breternitz, Carlo A.R. Perini, Armin Hoell, Pablo P. Boix, Susan Schorr, Joshua S. Kretchmer, Juan-Pablo Correa-Baena

**Affiliations:** † School of Materials Science and Engineering, Georgia Institute of Technology, Atlanta, Georgia 30332, United States; ‡ School of Chemistry and Biochemistry, Georgia Institute of Technology, Atlanta, Georgia 30332, United States; § National Synchrotron Light Source II, Brookhaven National Lab, Upton, New York 11973, United States; ∥ Department of Structure and Dynamics of Energy Materials, Helmholtz Zentrum Berlin für Materialien und Energie, Hahn-Meitner-Platz 1, 14109 Berlin, Germany; ⊥ Institut de Ciència dels Materials, Universidad de València, C/J. Beltran 2, Paterna 46980 Valencia, Spain; # Freie Universitaet Berlin, Institute of Geological Sciences, Malteser Str. 74-200, 12249 Berlin, Germany

## Abstract

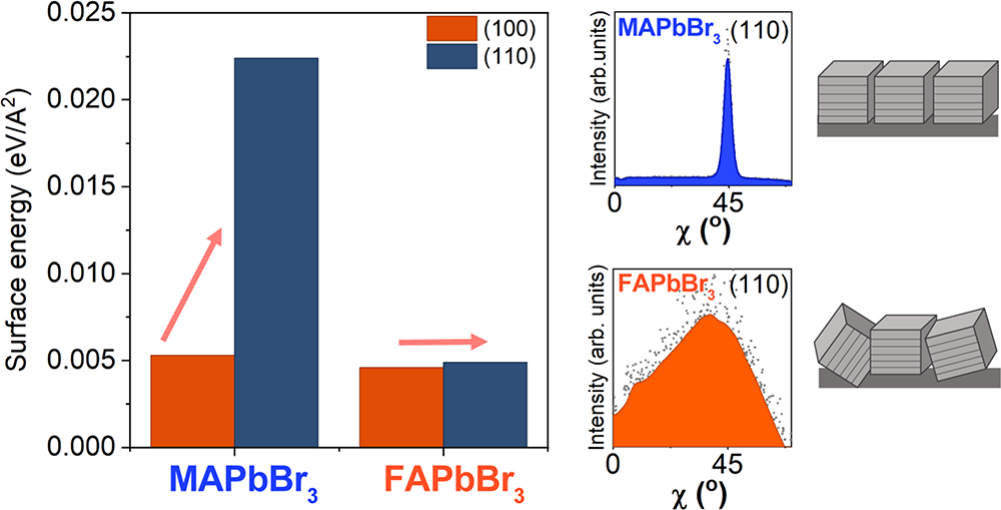

Preferred crystallographic orientation in polycrystalline films is desirable for efficient charge carrier transport in metal halide perovskites and semiconductors. However, the mechanisms that determine the preferred orientation of halide perovskites are still not well understood. In this work, we investigate crystallographic orientation in lead bromide perovskites. We show that the solvent of the precursor solution and organic A-site cation strongly affect the preferred orientation of the deposited perovskite thin films. Specifically, we show that the solvent, dimethylsulfoxide, influences the early stages of crystallization and induces preferred orientation in the deposited films by preventing colloidal particle interactions. Additionally, the methylammonium A-site cation induces a higher degree of preferred orientation than the formamidinium counterpart. We use density functional theory to show that the lower surface energy of the (100) plane facets in methylammonium-based perovskites, compared to the (110) planes, is the reason for the higher degree of preferred orientation. In contrast, the surface energy of the (100) and (110) facets is similar for formamidinium-based perovskites, leading to lower degree of preferred orientation. Furthermore, we show that different A-site cations do not significantly affect ion diffusion in bromine-based perovskite solar cells but impact ion density and accumulation, leading to increased hysteresis. Our work highlights the interplay between the solvent and organic A-site cation which determine crystallographic orientation and plays a critical role in the electronic properties and ionic migration of solar cells.

## Introduction

Preferred crystallographic orientation in polycrystalline films is highly desired for efficient charge carrier transport in organic–inorganic lead halide perovskite solar cells. Oriented crystallographic domains have been shown to enhance charge carrier transport^1^ and suppress ionic migration^2^ in lead halide perovskite films. Therefore, manipulating the degree of crystallographic orientation presents an important design principle.^3−5^ Furthermore, organic–inorganic lead bromide perovskites, compared to the more common lead iodide perovskites, have also shown potential for multijunction perovskite solar cells because of their wide bandgap and phase stability.^6−8^ Beyond solar cells, lead bromide perovskites have been used for light-emitting devices^9^ and photodetectors.^10,11^ For these reasons, understanding the structure–property relationships of bromine-based perovskites is fundamental to designing better optoelectronic devices. In particular, the underlying mechanisms that dominate crystallographic orientation in lead bromide perovskites are underexplored.

Solvent engineering has been used to control the crystallization kinetics and properties of perovskite polycrystalline thin films.^11−16^ The highest power conversion efficiency in perovskite solar cells has been through solution processing utilizing a precursor solution of a mixture of *N*,*N*-dimethylformamide (DMF) and dimethyl sulfoxide (DMSO).^12^ Differences in coordination between the solvent, DMF or DMSO, with the perovskite precursors salts (e.g., MABr, FABr, PbBr_2_) have led to variations in the crystallization process, affecting the film morphology and crystallographic orientation.^16−18^ Solvent coordination influences the formation of colloidal particles in solution, which has an important effect on the overall crystallization process and perovskite thin film morphology.^19−21^ For example, adding an optimum amount of DMSO slows down crystallization and helps control crystallinity in halide perovskites.^12,14,22,23^ Instead, DMF coordinates less strongly, leading to a faster and poorly controlled crystallization.^14,23^ The colloids in the precursor solution may agglomerate, forming colloidal particles and intermediate solvent-perovskite phases, all of which have an effect on the crystallized film.^19,20,24^ In particular, iodine-based lead perovskites form numerous types of iodo-plumbate structures in solution and other solvent-perovskite phases both in DMF and DMSO.^15,18,25^ Ray et al. studied and observed important differences in the precursor solution colloids leading to different final structures in Cs–Pb–Br complexes.^21^

In lead bromide perovskites, APbBr_3_, the A site can be the inorganic Cs atom or the organic methylammonium (MA) or formamidinium (FA) molecules.^1,3−5,26^ The A-site cation has also been shown to play a role in the crystallization behavior of halide perovskite thin films. For example, Petrov et al. showed that FA and MA produce different solvent-halo-plumbate complexes. For the Br-based compositions, they showed that the combination of MABr and PbBr_2_ in DMF or DMSO did not form any solvent intermediate phase as it converted directly into MAPbBr_3_ perovskite.^18^ However, in the case of FA, combining FABr and PbBr_2_ in DMSO, a solvent intermediate phase (FA)_2_PbBr_4_-DMSO was formed.^11,18^ This suggests that the A-site cation has a very strong influence on the crystallization of thin films. An et al. studied the Cs-FA iodide-bromide compositional space from a pure DMSO precursor solution, showing that the film becomes more oriented when Cs and Br were added.^27^ In agreement with An, Steele et al.^26^ studied the crystallographic orientation of all-inorganic lead iodide perovskites, where they found that incorporating Br into CsPbI_3_ reduced the orthorhombic lattice distortion and led to energetically favored preferred crystallographic orientation. Zheng et al.^3^ manipulated the crystallographic orientation of lead halide perovskites by incorporating alkali metal cations such as Cs and Rb into the A-site. These different studies have shown that both solvent and composition will influence the orientation. Further, separating the solvent and composition (A-site cation) effects is important to understand the dominating factors that lead to a high degree of orientation for an optimal material design.

Herein, we investigate the role of the solvent and of the organic A-site cation on controlling crystallographic orientation in lead bromide perovskites. To understand polycrystalline film orientation, we start from the early stages of crystallization by analyzing the precursor solutions through small-angle X-ray scattering (SAXS). We study the two organic cations MA and FA, both in DMF and DMSO, to unravel the A-site cation effect on orientation. Crystallographic film orientation is analyzed by grazing-incidence wide-angle X-ray scattering (GIWAXS). We observe an interplay between solvent and A-site cation that dominates the crystallographic orientation in lead bromide perovskites. We observe that DMSO creates a highly oriented film, compared to a more random orientation, when using DMF. For the A-site cation, regardless of the solvent, MAPbBr_3_ shows a higher degree of orientation than FAPbBr_3_. By calculating the surface energy of the (100) and (110) plane facets, we see that MAPbBr_3_ is thermodynamically favored to have the (100) planes oriented parallel to the surface; instead, FAPbBr_3_ exhibits a near degeneracy between the (100) and (110) facets, leading to a lower degree of preferred orientation. In addition, we show that the A-site cation, given differences in preferred orientation, does not affect ionic diffusion but does change the ionic density as a function of a bias, as shown in impedance spectroscopy. The latter causes increased hysteresis in solar cells. Our findings provide valuable guidelines for designing lead bromide perovskites with an optimum degree of preferred crystallographic orientation for optimal performance.

## Methodology

### Experimental Section

#### Perovskite Precursor Solution

We prepared a 1.24 M A-PbBr_3_ perovskite precursor solution with A being MA or FA by mixing MABr (Dyenamo, 99.99%) or FABr (Dyenamo, 99.99%) with PbBr_2_ (TCI, 99.99% purity). The stoichiometry of the solution had a 5% molar excess of the organic cation. The solvent used was DMF (Sigma, anhydrous 99.80%), DMSO (Sigma, anhydrous 99.80%), or a volume ratio of 4:1 DMF to DMSO. The solution was stirred for 1 h at 600 rpm before deposition in a nitrogen glovebox. The solution was kept in nitrogen before any characterization at room temperature. Pb-containing solutions are hazardous; therefore, we properly sealed the solutions in glass vials and handled them with butyl gloves inside the glovebox.

#### Perovskite Thin Film Deposition

The lead bromide perovskite precursor solution (80 μL) was spin-coated statically on an indium-doped tin oxide (ITO)/glass or glass substrate in a two-step program: first at 1000 rpm for 10 s (acceleration of 1000 rpm/s) and then at 6000 rpm for 20 s (acceleration of 2000 rpm/s). During the spin-coating process, 250 μL of chlorobenzene (CB, Sigma 99.50%) was added dynamically. For the perovskites in DMF, CB was added 15 s after the start of the spinning process, whereas for perovskites in DMSO, CB was added at 25 s. After deposition of the perovskite solution, the substrates were annealed at 100 °C for 30 min in a nitrogen glovebox.

#### Perovskite Solar Cells

The solar cells fabricated were of n-i-p architecture: fluorine-doped tin oxide (FTO)/compact TiO_2_/mesoporous TiO_2_/lead bromide perovskite/2,2′,7,7′-tetrakis(*N*,*N*-di-*p*-methozyphenyl-amine)9,9′-spirobifluorene (Spiro-OMeTAD)/Au. The compact TiO_2_ was deposited by spray pyrolysis, the mesoporous TiO_2_ by spin-coating, the Spiro-OMeTAD by spin-coating, and the Au by thermal evaporation. The experimental details of deposition and fabrication are found in our previous work.^1^

### Theoretical Calculations

The surface energies, γ, of FAPbBr_3_ and MAPbBr_3_ with different surface orientations were calculated using the following definition:
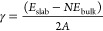
1where *E*_slab_ is the total energy the slab, *E*_bulk_ is the energy of a unit cell in the bulk of the film, *N* is the number of formula units within the slab, and *A* is the area of the surface. A slab is given by a finite number of layers of the material system that are fully periodic in *x* and *y* and isolated from a periodic image in the *z* direction by a vacuum gap. *E*_slab_ and *E*_bulk_ were computed with periodic DFT using Quantum Espresso.^28,29^ Pseudopotentials were given by the projector-augmented wave (PAW) method and used with the Perdew–Burke–Ernzerhof functional in the generalized gradient approximation. The kinetic energy cutoff for the wavefunction was set to 80 Ry, and a corresponding energy cutoff for the charge density was set to 800 Ry. A Monkhorst–Pack *k*-point grids of 4 × 4 × 4 and 4 × 4 × 1 were chosen for the Brillouin zone sampling of the bulk and the slab calculations, respectively. The super-cell for the bulk calculation consisted of a single unit cell. Variable cell optimization was performed on each bulk unit cell prior to the surface construction. Each supercell for the surface calculations was constructed with a 30 Å thick slab and 15 Å vacuum gap. A geometry optimization with a fixed cell volume and shape was performed for each slab. All slabs were constructed with a complete number of perovskite formula units, resulting in two different terminations for the bottom and top surfaces. The surface energy for each termination was computed by freezing one of the surfaces in accordance with the procedure described previously.^30^ One surface layer of the inorganic substructure composed of 1 Pb and 3 Br atoms was frozen in each slab, while the nuclei of the opposing surface were allowed to relax freely. All cations were set to rotate freely to account for the presence of the variety of cation orientations in the structure and its mobile nature. Due to the asymmetry introduced by the cation structure to the unit cell, cation orientation relative to the inorganic lattice and the surface was shown to break the degeneracy within a single family of plains.^30^ In this study, we use the lowest energy surfaces as representative structures of the {100} and {110} families of planes. The chosen cation orientations also agree with the structures reported in the literature.^30,31^ The converged geometries for each surface calculation are provided in the Supporting Information (SI).

### Characterization

#### SAXS

The SAXS measurements were performed on lead bromide solutions using the HZB ASAXS instrument^32^ installed at the four-crystal monochromator beamline (FCM) of the Physikalisch-Technische Bundesanstalt (PTB)^33^ and operated at the BESSYII synchrotron of Helmholtz-Zentrum Berlin für Materialien und Energie (HZB). The solutions were measured in transmission in flat rectangle-shaped capillaries of 0.1 mm thickness under vacuum conditions using monochromatic X rays of 10 keV. We used a four-crystal monochromator with Si 111 crystals. This energy was optimized for the sample’s transmission and scattering vector range. Data were collected three times for each sample with 600 s illumination per image. The images did not show any notable differences, implying that the solutions were stable under the chosen conditions. The 2D scattering patterns were azimuthally integrated and corrected for instrumental background and contributions of the sample holder through the BerSAS software.^34^

#### GIWAXS

The GIWAXS measurements were performed at the beamline for Complex Materials Scattering (11-BM) at the Brookhaven National Laboratory. The perovskite films for GIWAXS characterization were deposited on ITO/glass. An X-ray beam (13.5 keV, λ = 0.918 Å) with a footprint of 0.2 mm (height) × 0.05 mm (width) was irradiated on samples in vacuum (∼10^–5^ torr) for 10 s with an incidence angle of 0.5°. Beam divergence was 1 mrad and energy resolution 0.7%. The data were analyzed using the SciAnalysis package provided by the beamline.

#### Photovoltaic Performance

The current density–voltage (*J–V*) characteristics of the solar cells were measured using a LITOS LITE setup (Fluxim, Switzerland), equipped with a Wavelabs Sinus-70 AAA solar simulator with standard AM1.5 G illumination at room temperature and ambient air. The *J–V* curves were obtained by scanning voltage in the range from 1.4 to −0.5 V with a scan speed of 50 mV·s^–1^ first in reverse and then in forward scan directions. The active area of the device was 0.128 cm^2^, and a black metal mask with an aperture area of 0.0625 cm^2^ was used to define the illuminated area.

#### Impedance Spectroscopy (IS)

IS was carried out using a PAIOS hardware (Fluxim, Switzerland) on complete solar cells at room temperature under one sun illumination and in ambient air. The measurements were performed at five different offset voltages spaced from 0 V to the open circuit. The sweep frequency was varied from 10 MHz to 0.1 Hz, with an amplitude of 10.0 mV. Z-view software was employed to analyze the results and fit the data to the equivalent circuit.

## Results and Discussion

### Early Stages of Crystallization

**Figure 1:**
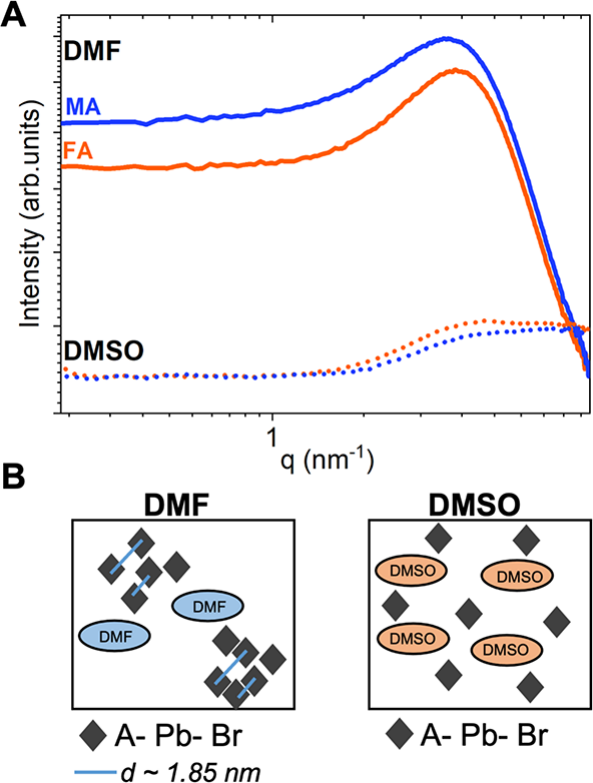
Particle interactions in the precursor solution. (A) SAXS data of the four studied systems MAPbBr_3_ in DMF or DMSO and FAPbBr_3_ in DMF or DMSO. (B) Schematic of the particle interaction in DMF and DMSO interpreted from SAXS data, A is MA or FA, and *d* is the interparticle distance.

To analyze the perovskite precursor solutions, we measured SAXS of MABr-PbBr_2_ and FABr-PbBr_2_ precursors in DMF and DMSO (1 M). SAXS is a technique widely used to analyze the structure of nanoparticles in solution and their colloidal properties and reveals information about size, distribution, and the interaction of the particles.^20,35^ Flatken et al. introduced SAXS as a technique to reveal the colloidal nature of lead halide perovskites.^20^ Moreover, SAXS allows for the analysis of high concentration solutions in comparison to other techniques such as dynamic light scattering (DLS) and UV–VIS spectroscopy, which require lower concentrations.^19,20,36^Figure 1 shows SAXS results and interpretation. In Figure 1A, the DMF solutions generate clear and strong scattering peaks. In SAXS patterns, the scattering intensity indicates the presence of particles in solution. In addition, the scattering peak maximum results from particle interactions in solution.^20^ This strong particle interaction indicates a quasi-crystalline prearrangement of particles in solution, which will affect the early stages of crystallization.^20,37^ The maximum peak position at *q*_max_ reveals the mean *d* spacing from Bragg’s law *d* = 2π/*q*_max_. The *d* spacing is the distance between the mass centers of interacting colloidal particles. From Figure 1A, we calculate the *q*_max_ from a Lorentzian fitting and retrieve the *d* spacing (fitting in Figure S1). For both A-site cations in DMF, there is colloidal particle interaction in solution. The calculated interparticle distance for both FA and MA particles is around 1.85 nm. Flatken et al. have shown a similar SAXS peak when preparing different lead iodide perovskites such as MAPbI_3_ and FAPbI_3_ in a mixture of DMF and DMSO.^20,24^ However, the pure DMSO precursor solution did not show a clear scattering peak (Figure 1A). In comparison to DMF, DMSO prevents the A–Pb–Br interaction as DMSO binds more strongly than DMF to the precursor salts, preventing the A–Pb–Br interaction. This suggests that there is no colloidal particle interaction. In contrast, in DMF, a solvent with weaker coordination, the colloidal particles interact with each other and form clusters.^16^ This is in agreement with the previous work by Ray et al., which showed that due to the strong coordination of DMSO, the particles in solution do not interact with each other, remaining isolated.^21^ Their work also showed that DMSO forms colloidal particles one order of magnitude smaller than in DMF.^21^ The schematics in Figure 1B show the hypothesized differences in particle interaction for DMF and DMSO. The shape and composition of these particles are unknown but based on the work of Ray et al.^21^ and Flatken et al.,^24^ we speculate that these are bromo-plumbate octahedra, the result of the interaction between A–Pb–Br in solution. We observe that in the bromine-based precursor solution, like in iodine-based perovskites,^14,16^ the colloidal particle interactions in solution are driven by the solvent, while the A-site cation does not appear to play a role in the colloidal interactions.

### Preferred Crystallographic Orientation

**Figure 2:**
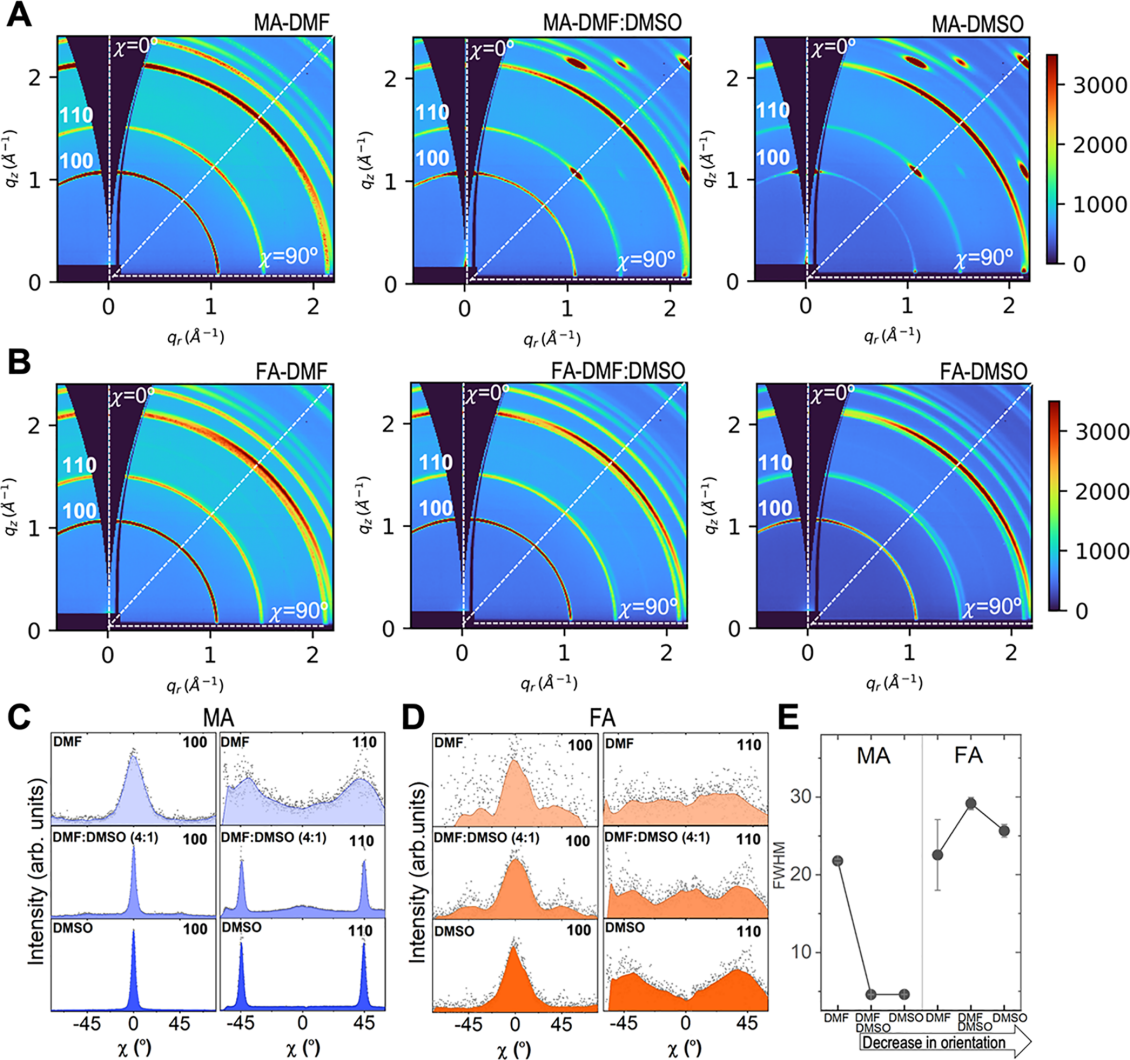
Interplay of solvent and A-site cation in the crystallographic orientation of lead bromide films studied by GIWAXS. (A, B) 2D GIWAXS patterns in DMF, DMF:DMSO (4:1 volume), and DMSO for (A) MAPbBr_3_ and (B) FAPbBr_3_. (C, D) Azimuthal integration profiles of the main Debye–Scherrer rings (100 and 110) as a function of the χ angle from GIWAXS for three solvent systems for (C) MAPbBr_3_ and (D) FAPbBr_3_. (E) FWHM and error bar of the 100 azimuthal peak for MAPbBr_3_ and FAPbBr_3_ as a function of the solvent.

We performed GIWAXS measurements on thin films prepared with DMF and DMSO to examine the effect of different cluster formations arising from the different solvents on the orientation of the thin films. Synchrotron-based GIWAXS has been widely used to analyze the crystallographic orientation and the structure of the lead halide perovskites in thin films and solar cells.^26,38^ First, we study the effect of the solvent of the precursor solution on the crystal phases and crystallographic orientation by comparing the deposition of FAPbBr_3_ and MAPbBr_3_ in DMF, DMF:DMSO (4:1 volume), and DMSO. The 2D GIWAXS patterns were integrated to provide 1D diffraction patterns that are shown in Figure S2. For all the solvents, MAPbBr_3_^39^ and FAPbBr_3_^40,41^ display diffraction peaks corresponding to a cubic symmetry *Pm*3̅*m*. To identify crystallographic orientations, we analyzed the Debye–Scherrer rings from the GIWAXS patterns in Figure 2_._Figure 2A shows the 2D GIWAXS for MAPbBr_3_. As we change the solvent from DMF to DMSO, we see a clear evolution of the crystallographic orientation. For DMF, we observe a complete Debye–Scherrer ring of the (100) plane at *q_r_* ∼ 1.06 ± 0.06 Å^–1^, which indicates random orientation as the crystallographic domains scatter X-rays in all angles. The complete ring sharpens into an arc when adding one-fourth of the volume of DMSO, showing a preferred orientation of the crystallographic domains. For pure DMSO, the arc of the (100) plane sharpens further into a high-intensity Bragg spot, indicating a high degree of preferred orientation. Figure 2B shows the 2D GIWAXS for FAPbBr_3_, where the evolution and degree of orientation differ. For DMF and DMF:DMSO, FAPbBr_3_ shows a uniform intensity in all the rings, suggesting that the film has no preferred orientation. For pure DMSO, the ring of the (100) plane sharpens into an arc with maximum intensity at *q_r_* = 0, indicating an increase in the degree of preferred crystallographic orientation from random to preferentially oriented crystallographic domains.

To further analyze and quantify crystallographic orientation, we integrated the azimuthal profile (χ) of the Debye–Scherrer rings of the first two peaks corresponding to the (100) plane at *q_r_* ∼ 1.06 ± 0.06 Å^–1^ and the (110) plane at *q_r_* ∼ 1.4 ± 0.06 Å^–1^. The peak of the azimuthal profile provides information about the direction of the preferred orientation of the planes. Since the grazing-incidence measurements give rise to a missing wedge near the *q_z_* axis due to the curvature of the Ewald sphere, it is important to analyze the orientation of the two planes. We studied the (100) and (110) planes, where the (110) plane complements the quantitative description of orientation. The (100) and (110) Debye–Scherrer rings are integrated from χ = −80° to 80°, where χ = 0° is set at *q_r_* = 0 (out-of-plane) and χ = 90° at *q_z_* = 0 (in-plane). For MAPbBr_3_, we integrated the azimuthal profile of the Debye–Scherrer rings from Figure 2A into Figure 2C, and for FAPbBr_3_, the azimuthal profiles are integrated from Figure 2B into Figure 2D. To understand the dispersion in the preferred orientation of the crystallographic domains, we fit the azimuthal profile peaks into a pseudo-Voigt or Gaussian function from which we obtain the full width at half-maximum (FWHM) and other statistical parameters shown in Table S1. The azimuthal profile for MAPbBr_3_ in Figure 2C shows a peak at 0° of the (100) plane, evidence that these planes are oriented parallel to the substrate. In addition, the azimuthal profile peak of the (110) plane is around 45°, which is consistent with the (100) plane being oriented parallel to the substrate (explanation in Figure S3). We also observe that the choice of solvent changes the FWHM of the fitted azimuthal peak, as expected from the evolution in the degree of orientation seen in the 2D GIWAXS patterns in Figure 2A. In MAPbBr_3_ in DMF, the (100) peak is broader, and the fitting has a larger error and low *R*^2^ (Table S1). When DMSO is added (DMF:DMSO and pure DMSO), the azimuthal peaks sharpen, and the raw data fits nicely into the pseudo-Voigt function with a high *R*^2^ (Table S1). The sharp azimuthal profile peaks indicate a high degree of preferred orientation of the (100) plane parallel to the substrate. In addition, the MAPbBr_3_ from DMSO has the lowest FWHM, hence the highest degree of preferred orientation.

In Figure 2D, we plot the azimuthal profile peaks for FAPbBr_3_. Compared to MAPbBr_3_, the FAPbBr_3_ peaks are all broader and have a higher FWHM, showing a lower degree of preferred orientation. For DMF, the crystallographic domains show dispersion in the integrated data, suggesting a low degree of preferred orientation. The fitted Gaussian peak for DMF has a very low *R*^2^ and large error in Table S1. The poor fitting for FAPbBr_3_-DMF is attributed to the random orientation of the film. As we added DMSO to the precursor solution, the *R*^2^ of the fit of the (100) azimuthal peak 0° increased, showing a preferred orientation of crystallographic domains with the (100) plane parallel to the substrate. For FAPbBr_3_-DMF:DMSO, the (110) azimuthal profile shows three broad peaks at around ± 45° and 0°, which indicates a lower degree of orientation and a higher dispersion. With pure DMSO, the (100) azimuthal peak sharpens, the (110) azimuthal profile shows two broad peaks at ± 45°, and the data fitting improves, with a higher *R*^2^ and lower error in Table S1. These changes suggest that pure DMSO induces some preferred orientation, even in FA-based films. These results on preferred crystallographic orientation were corroborated by comparing the ratio in intensity between the 100 and 110 peaks from Bragg Brentano XRD to complement the missing wedge from GIWAXS (Figure S4). The FWHM of the (100) azimuthal peak shown in Figure 2E quantifies the dispersion of orientation of the crystallographic domains. MAPbBr_3_ films deposited from a pure DMSO precursor solution show the lowest FWHM, evidence of the highest degree of preferred orientation. In contrast, FAPbBr_3_ shows a higher FWHM, decreasing the degree of preferred orientation. Additionally, we verified that the preferred orientation was induced by the solvent and was not kinetically determined by the antisolvent addition times. Figures S5 and S6 show the GIWAXS patterns and azimuthal integration of lead bromide perovskite films adding CB as antisolvent at different times during the spin-coating process.

The SAXS results discussed in the previous section suggest that there are interactions between the bromo-plumbate octahedra colloidal particles in DMF, which favor the possible formation of agglomerates in solution. We speculate that these colloidal aggregates could form dispersed and agglomerated nuclei for the crystallization process. This could lead to the random orientation observed in DMF from the GIWAXS measurements, as illustrated in the schematics of Figure S7. The higher degree of random orientation in DMF is observed regardless of the cation type, indicative of this solvent-dominated phenomenon. To further understand the origins of preferred crystallographic orientation in lead bromide perovskites, we now discuss why MA and FA impact preferred orientation in pure DMSO, where there are no colloidal interactions. Instead, in DMSO, the octahedra particles and nuclei would be isolated, and the crystallization is slowed down. We also learned from SAXS in Figure 1 that there were no differences between MA and FA in solution. However, the crystallized films in pure DMSO still show differences in orientation when comparing MAPbBr_3_ and FAPbBr_3_. For this reason, other underlying forces and mechanisms must lead to these differences in orientation for the different A-site cations in lead bromide perovskites.

### Mechanisms for Preferred Orientation

**Figure 3:**
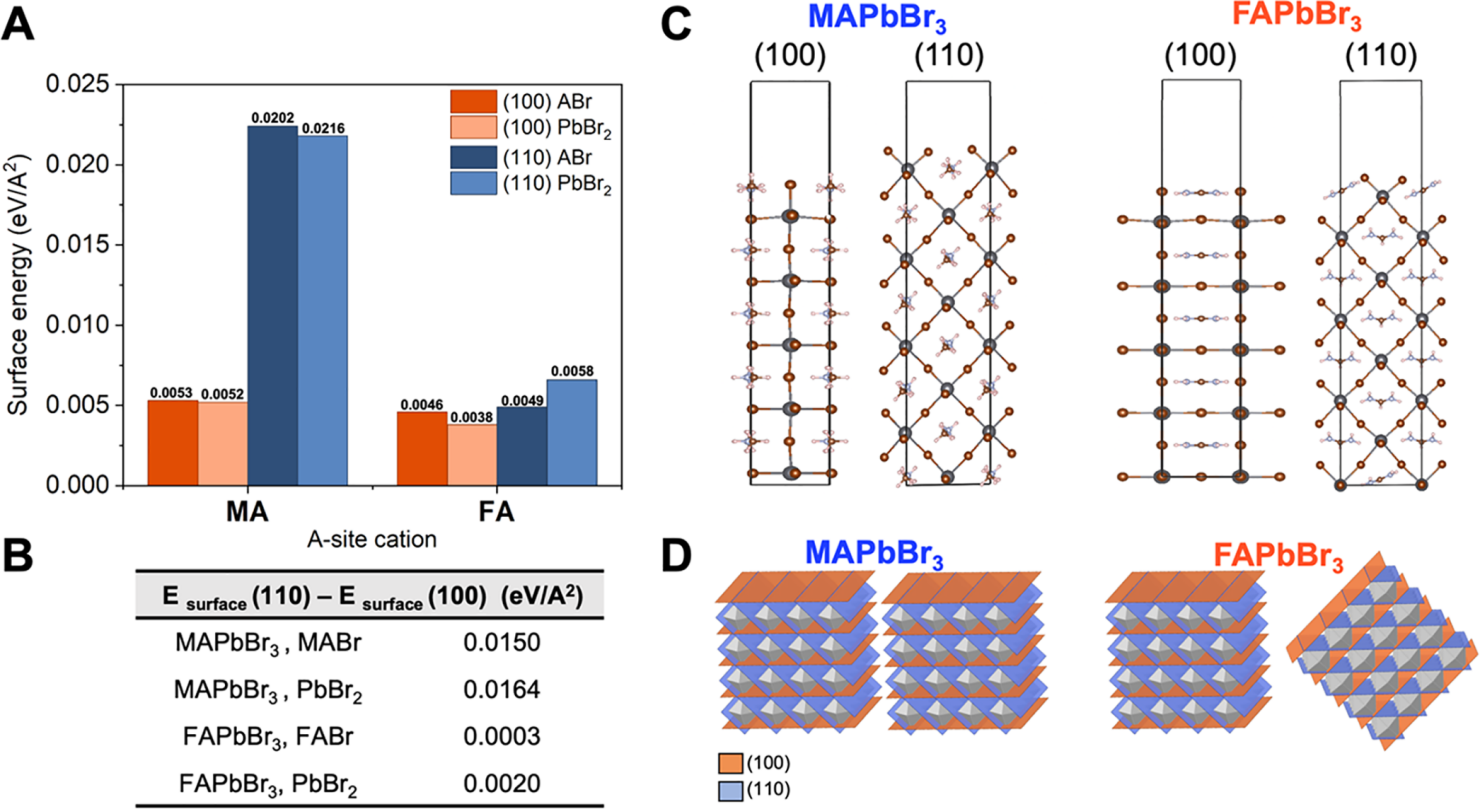
Results of the DFT calculation for the representative (100) and (110) surfaces of MAPbBr_3_ and FAPbBr_3_. (A) Surface energy of the analyzed structures computed for surfaces with PbBr_2_ or FABr/MABr termination. (B) Energy difference between the corresponding (110) and (100) surfaces. (C) Representative final structures of the FABr/MABr-terminated calculation for MAPbBr_3_ and FAPbBr_3_. The inorganic lattice of the bottom surface demonstrates the PbBr_2_ phase was frozen throughout the calculation. (D) Pictorial representation of the increased surface disorder due to the presence of two competing surfaces in FAPbBr_3_.

We used periodic DFT to calculate the relative surface energies for MAPbBr_3_ and FAPbBr_3_ to help explain the difference in crystallographic orientation observed in the GIWAXS measurements arising from the difference in A-site cation. The calculated surface energies for a representative surface from the {100} and {110} family of planes are presented in Figure 3A. We considered both PbBr_2_ and FABr/MABr termination, but we observed that the termination only leads to a qualitatively negligible variation in the surface energy regardless of A-site cation and surface plane. Therefore, the following qualitative conclusions are discussed regardless of this distinction.

Figure 3A shows that the (100)-terminated slab is energetically more stable in comparison to the (110)-terminated slab for MAPbBr_3,_ in agreement with previous calculations. The (100) and (110) FAPbBr_3_ have almost equivalent surface energies. The energy differences between the (110) and (100) surfaces for both cations are presented in Figure 3B. The large energy difference for MA compared to FA agrees with and helps explain the experimentally observed difference in crystallographic orientation. For MA, the (100) surface is energetically more stable and will therefore be the dominant surface that is formed. For FA, the relative degeneracy between the (100) and (110) surfaces implies no thermodynamic driving force to form one surface over the other. Therefore, both surfaces can be easily formed, leading to a higher degree of orientational disorder.

The relative difference in surface energies between the MAPbBr_3_ and FAPbBr_3_ surface can be understood by comparing the orientation of the A-site cation in the final surface structures (Figure 3C) to that in the bulk. In all cases, the organic A-site cation adopts a configuration where its dipole moment is parallel to the surface to minimize the energetic penalty of forming the surface. The difference is that in MAPbBr_3_, the orientation of the A-site cation in the bulk already corresponds to a planar configuration; MAPbBr_3_ only exhibits a minor geometric reorganization for both the (100) and (110) surfaces in comparison to the bulk structure associated with a rotation along the C–N bond for the MA cation. Similarly, the FA cation is already planar in the (100) surface and therefore does not reorient significantly. However, the bulk configuration of the FA cation is perpendicular to the (110) surface plane. Therefore, the FA cations close to the surface significantly reorient upon the formation of the (100) surface, such that they adopt a configuration parallel to the (100) surface. This reorientation leads to an energetic stabilization of the (100) surface and results in the relative energy degeneracy between the (100) and (110) terminations. This planar configuration of the FA cation has been previously observed in the context of FAPbI_3_.^42^ While this analysis does not account for the kinetics of surface formation, the presence of two competing surface orientations in FAPbBr_3_ provides thermodynamic insight into the increased disorder observed in the GIWAXS measurements (Figure 3D).

### Solar Cell Performance and Ionic Movement

**Figure 4:**
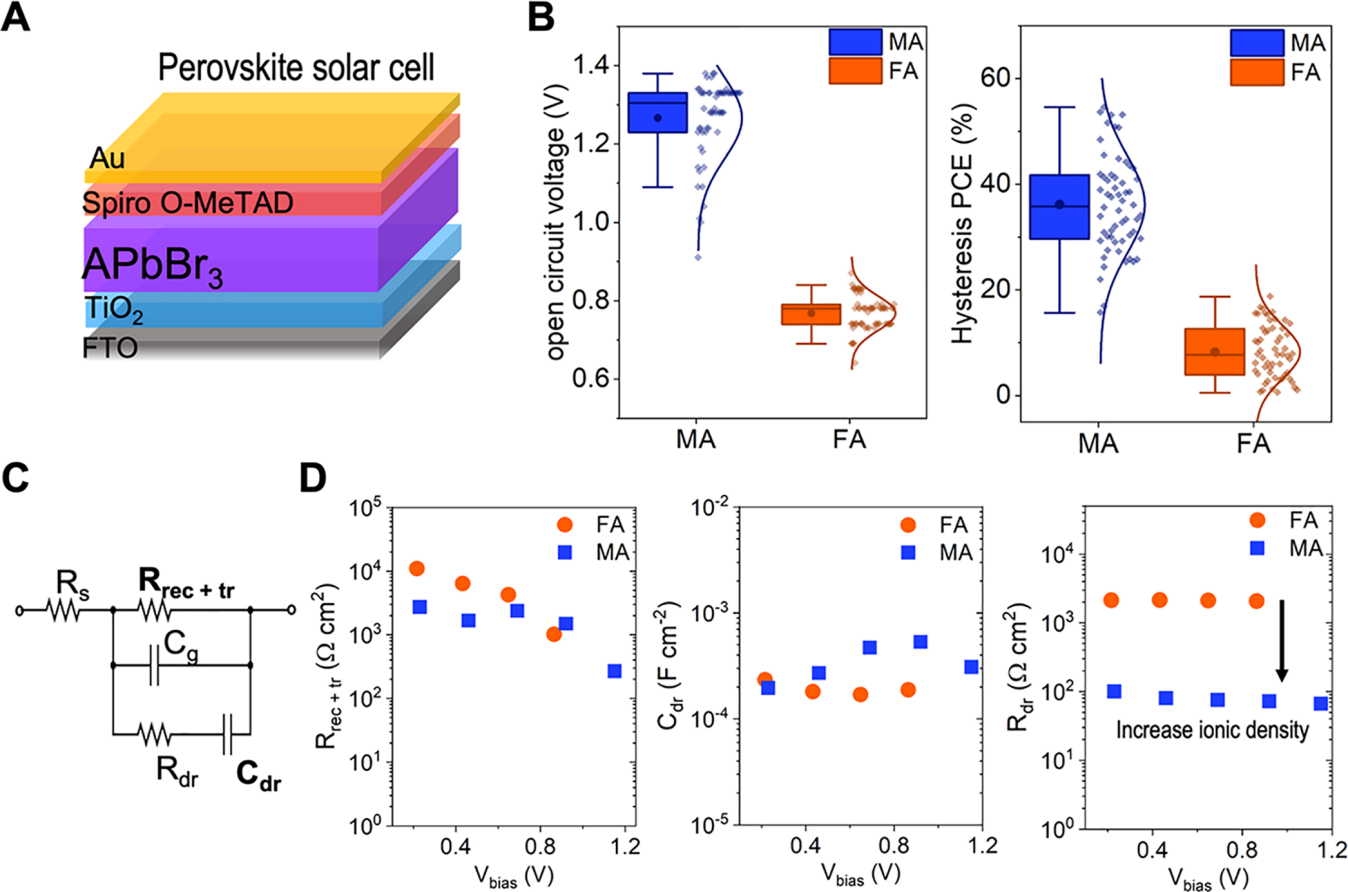
Electrical properties of the solar cell. (A) Schematic of a perovskite solar cell architecture where A in APbBr_3_ is MA or FA. (B) Box plots of the solar cell characterization from the *J*–*V* curve open circuit voltage, and absolute hysteresis index of the power conversion efficiency (PCE). (C, D) Impedance spectroscopy measurements for MAPbBr_3_ and FAPbBr_3_ solar cells. (C) Equivalent circuit used for the analysis of the impedance spectra. (D) Recombination and transport resistance for different applied biases (*R*_rec+tr_), low-frequency capacitance for different applied biases (*C*_dr_), and low-frequency resistor (*R*_dr_).

Having understood the effect of the A-site cation on preferred crystallographic orientation, we studied how the choice of A-site cation impacts the electric response in perovskite solar cells. We confirmed the same differences in the preferred orientation of the MA and FA lead bromide perovskites on the solar cell substrate (Figure S8). The solar cells had an n-i-p architecture that is described in the experimental section, and is shown schematically in Figure 4A. Lead bromide perovskite solar cells exhibit significantly lower efficiency compared to their iodine counterparts due to their wider bandgap and unoptimized contacts.^43−45^ However, their large bandgap expands the scope of application into multijunction solar cells or other optoelectronic applications. We measured the *J*–*V* characteristics of the solar cells under simulated solar illumination (AM 1.5 G, 100 mW/cm^2^) to calculate the figures of merit of the solar cells (*J*–*V* curves example in Figure S9). We measured the open circuit voltage, short circuit current, fill factor, power conversion efficiency (PCE) from the *J*–*V* scan, and the stabilized PCE from the maximum power point tracking (PCE_MPPT_). The statistical distribution of the open circuit voltage is shown in Figure 4B. The optical bandgap variations (Figure S10) dominate the trends in the open circuit voltage. A larger bandgap for MA relative to FA increases the open circuit voltage and decreases the short circuit current (Figure S11). Given the differences in the bandgap, we cannot attribute the differences in charge carrier transport solely to crystallographic orientation. However, based on previous studies, a better charge carrier transport is expected for an oriented polycrystalline thin film.^1,3^ The other figures of merit of the solar cells, fill factor, and PCE from the reverse scan are reported in the SI (Figure S11).

Beyond the effect of crystallographic orientation on charge carrier transport in perovskite solar cells, some studies have shown that orientation can influence the pathway of ionic movement. It has been suggested that the halide and Pb ions can move along the (110) plane.^2^ Hysteresis of the *J*–*V* curve in lead halide perovskite solar cells has been attributed to ion migration,^46^ among others.^47−49^ Although ionic movement is not solely associated with *J*–*V* curve hysteresis,^50^ we analyze the hysteresis behavior to get an insight into how crystallographic orientation influences ion migration. Therefore, we calculated the absolute hysteresis index from the PCE to study the effects of ionic movement by comparing the difference between the forward scan (FS) and reverse scan (RS) of the PCE as follows:^51^
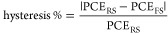
2

The devices made with FAPbBr_3_ perovskites show a lower hysteresis than those made with MAPbBr_3_. The hysteresis behavior associated with ion migration that is dependent on lead halide perovskite composition is in line with the previous reports.^47,49,52^ In MAPbBr_3_, the defect activation energy barrier for ion movement has been calculated to be lower than that of FAPbBr_3_, giving rise to more ionic movement, and explaining the larger hysteresis behavior for MAPbBr_3_.^52^ In addition, the hydrogen bond between the organic A-site cation and the bromine is stronger for FA than MA, limiting the motion of the cation in the case of FAPbBr_3_.^52^ This could be an additional explanation for the increase in hysteresis in MAPbBr_3_ solar cells.

To further understand the role of A-site cation on ionic motion in these materials, we measured impedance spectroscopy under different biases. The resulting Nyquist plots reproduce the characteristic patterns of lead halide perovskite solar cells^53^ (see representative spectra in Figure S12). The Nyquist plots were fitted to an equivalent circuit reported elsewhere (illustrated in Figure 4C).^54^ This circuit includes a resistor (*R*_rec*+*tr_) that couples both the recombination and transport resistances due to the low chemical capacitance of perovskite solar cells and a low-frequency branch with a capacitor (*C*_dr_) and resistor (*R*_dr_), both related to the ionic nature of the lead halide perovskites. In Figure 4D, *R*_rec*+*tr_ displays negligible variations between MAPbBr_3_ and FAPbBr_3_ under lower bias values.

High capacitance values at low-frequency regimes have been a fingerprint of lead halide perovskite solar cells.^54^ The origin of this low-frequency capacitance is typically attributed to the mixed ionic-electronic nature of these materials.^53,55^ In the equivalent circuit employed to analyze the results, this capacitance is modeled by a constant phase element *C*_dr_ to account for dispersive phenomena, yet with exponent values close to 1. The *C*_dr_ can be interpreted as an indication of increased ionic density or as higher ionic diffusion.^56^ Therefore, a comparative analysis of the *C*_dr_ is used to understand the ionic differences between MAPbBr_3_ and FAPbBr_3_, as observed in Figure 4D. FA has a slightly lower *C*_dr_ than MA. The slight reduction of *C*_dr_ can be interpreted as an indication of increased ionic diffusion or higher ionic density in FAPbBr_3_. To decouple the effects between ionic density and ionic diffusion, we examined the low-frequency part of the spectrum *R*_dr_. The increase of *R*_dr_ for FAPbBr_3_ compared to MAPbBr_3_ confirms the higher ionic density of the latter. These variations are in line with the increase of the absolute *J*–*V* hysteresis in MAPbBr_3_ solar cells (Figure 4B). These experimental results align with published computational results^52^ in which bromide vacancies and interstitials had much lower formation energies and higher densities in MAPbBr_3_ than for FAPbBr_3_, where the FA cation suppressed ion diffusion.

Analyzing the effect of preferred crystallographic orientation on the ionic effects, we observe that MAPbBr_3_, with a high preferential oriention, has the highest ionic density compared to the randomly oriented FAPbBr_3_. We suggest that ionic effects could be affected by the crystallographic orientation in lead bromide perovskites. Previous studies have shown the effects of in-plane crystallographic orientation of lead halide perovskites, where it has been observed that preferred orientation leads to a faster movement of ions. Fassl et al. studied the effect of in-plane crystallographic orientation on ionic transport rate from simulations and experimental work.^57^ They found that the relative orientation of the crystals affects ionic migration in polycrystalline films; crystals with preferred orientation had uniform ion transport while randomly oriented crystals had varying rates of ionic transport. Further, Eames et al. showed that in perovskite films with vacancy defects,^58^ the halide migrates along the octahedron edge between halide sites.^58^ Given that mobile ions can move in the ⟨110⟩ family of directions, Flannery et al.^2^ observed that ionic movement through the absorber layer is higher if the crystals in a mixed-halide perovskite film were highly oriented in the (110) plane. While an effect of orientation on charge transport cannot be excluded, we show that increased preferred orientation along the (100) plane does not contribute to an improved ion diffusion. Differences in the ionic contribution are ascribed to an increased ionic density in the highly ordered MAPbBr_3_ perovskite.

## Conclusions

We studied the effect of the solvent and organic A-site cation on the crystallographic orientation in lead bromide thin films. We showed that there is an interplay between the type of solvent and A-site cation that determines the preferred crystallographic orientation in bromine-based perovskites. Polycrystalline thin films prepared from solution in DMF solvent exhibit less preferred orientation, whereas films prepared from solutions in DMSO exhibit higher degree of preferred orientation for both FA and MA. Regardless of the solvent, MAPbBr_3_ showed the highest degree of preferred orientation compared to FAPbBr_3_. Theoretical calculations showed that MAPbBr_3_ is energetically favored to grow along the (100) plane while in FAPbBr_3_, the (100) and (110) surfaces are nearly degenerate in energy, and both are equally favored for growth. In addition, we observed that there are larger ionic movement effects in the highly oriented MAPbBr_3_ solar cells. The *J*–*V* hysteresis of the solar cells and impedance spectroscopy results indicate higher ionic density effects in MAPbBr_3_. This work provides new insights on the role of both solvent and A-site cation in the crystallization of thin films, crucial for long-term stability of perovskite solar cells.

## Supporting Information Available

Complete experimental section with materials and methods; iodide-based results; peak assignment for the Cs_0.17_FA_0.83_PbBr_3_ sample after humidity exposure; details on the azimuthal integration from GIWAXS; further SEM images and the complete optical properties values of all studied films; survey spectra and complete information on XPS; details about the TRMC setup and perovskite lifetimes; and impedance spectroscopy complete data (PDF)
Crystallographic information files for the studied compounds (ZIP)
