# Supplementary material for: Solvent and A-Site Cation Control Preferred Crystallographic Orientation in Bromine-Based Perovskite Thin Films
Source: Chem Mater. 2023 May 25;35(11):4181–91. doi: 10.1021/acs.chemmater.3c00075 (PMC10269330; doi:10.1021/acs.chemmater.3c00075)
Supplement: Supplementary file 1 — cm3c00075_si_001.pdf [file cm3c00075_si_001.pdf]

Supplementary Information

Solvent and A-Site Cation Control Preferred Crystallographic Orientation in Bromine-Based Perovskite Thin Films

Juanita Hidalgo [1], Yu An [1], Dariia Yehorova [2], Ruipeng Li [3], Joachim Breternitz [4], Carlo A.R. Perini [1], Armin Hoell [4], Pablo P. Boix [5], Susan Schorr [4,6], Joshua S. Kretchmer [2]*, Juan-Pablo Correa-Baena [1]*

[1] School of Materials Science and Engineering, Georgia Institute of Technology, Atlanta, Georgia 30332, United States.

[2] School of Chemistry and Biochemistry, Georgia Institute of Technology, Atlanta, Georgia 30332, United States.

[3] National Synchrotron Light Source II, Brookhaven National Lab, Upton, New York, 11973, United States.

[4] Department of Structure and Dynamics of Energy Materials, Helmholtz Zentrum Berlin für Materialien und Energie, Hahn-Meitner-Platz 1, 14109, Berlin, Germany.

[5] Institut de Ciència dels Materials, Universidad de València, C/J. Beltran 2, Paterna, 46980 Valencia, Spain.

[6] Freie Universitaet Berlin, Institute of Geological Sciences, Malteser Str. 74-200, 12249 Berlin, Germany.

Corresponding Authors: JSK jkretchmer@gatech.edu, and JPCB jpcorrea@gatech.edu

1. Experimental

SEM: The surface morphology of perovskites was characterized by scanning electron microscopy using a SEM Hitachi SU8010 operating at 5keV and 10μA.
Ultraviolet-visible absorption spectra (UV-Vis), photoluminescence (PL): UV-Vis was conducted using Cary 500 spectrometer (Varian, USA) in the 350–850 nm wavelength range at room temperature. PL was acquired with a Horiba FL3-2i spectrofluorometer. We measured UV-Vis and PL to the precursor solution and thin films deposited on glass substrates.
XRD: X-ray diffraction was measured using a Malvern PANalytical Empyrean with a Cu(kα) radiation of 1.54 Angstroms. The XRD patterns were collected in Bragg-Brentano geometry.

2. Results

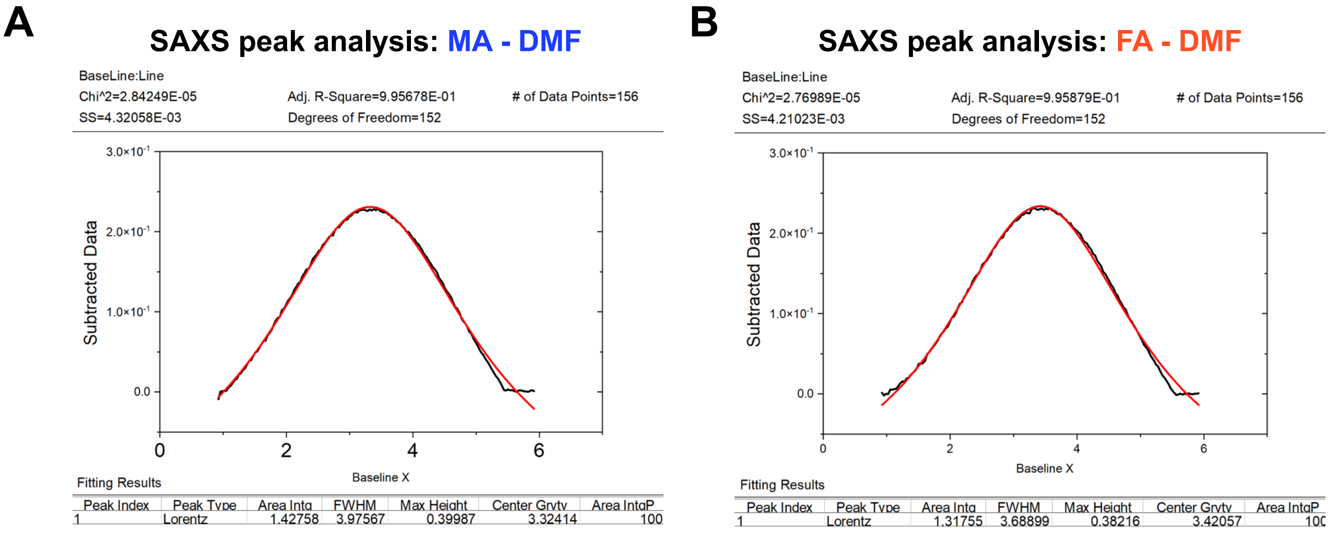

Figure S1. SAXS peak fitting for **(A)** $MAPbBr_3$ and **(B)** $FAPbBr_3$ in DMF (Lorentz function). The Integrated area, FWHM, maximum height, and center of the peak (Center Grvty) are shown.

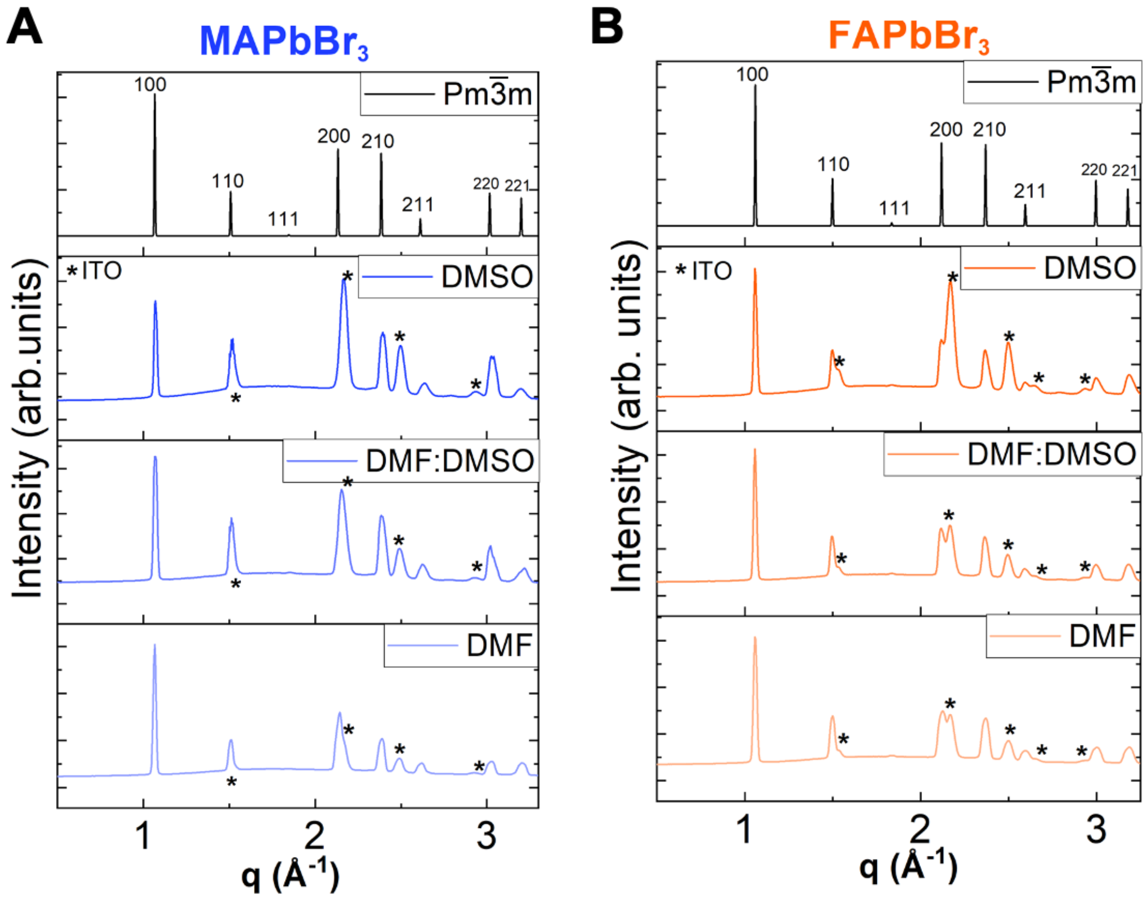

Figure S2. GIWAXS integrated circular average diffraction patterns for **(A)** MAPbBr3 and **(B)** $FAPbBr_3$ deposited from different solvents (DMF, DMF:DMSO 4:1 v/v, DMSO). The perovskite was deposited on glass/ITO substrates, and the * indicates the ITO diffraction peaks.

Note: Figure S2 shows the 1D GIWAXS diffraction patterns. The ITO substrate plays an important role since the main ITO diffraction peaks overlap with the 110 and 200 peaks of $MAPbBr_3$ and $FAPbBr_3$. It is important to know that there is no peak split but an overlap of the main ITO peak with the perovskite, and similar happens with other ITO peaks.

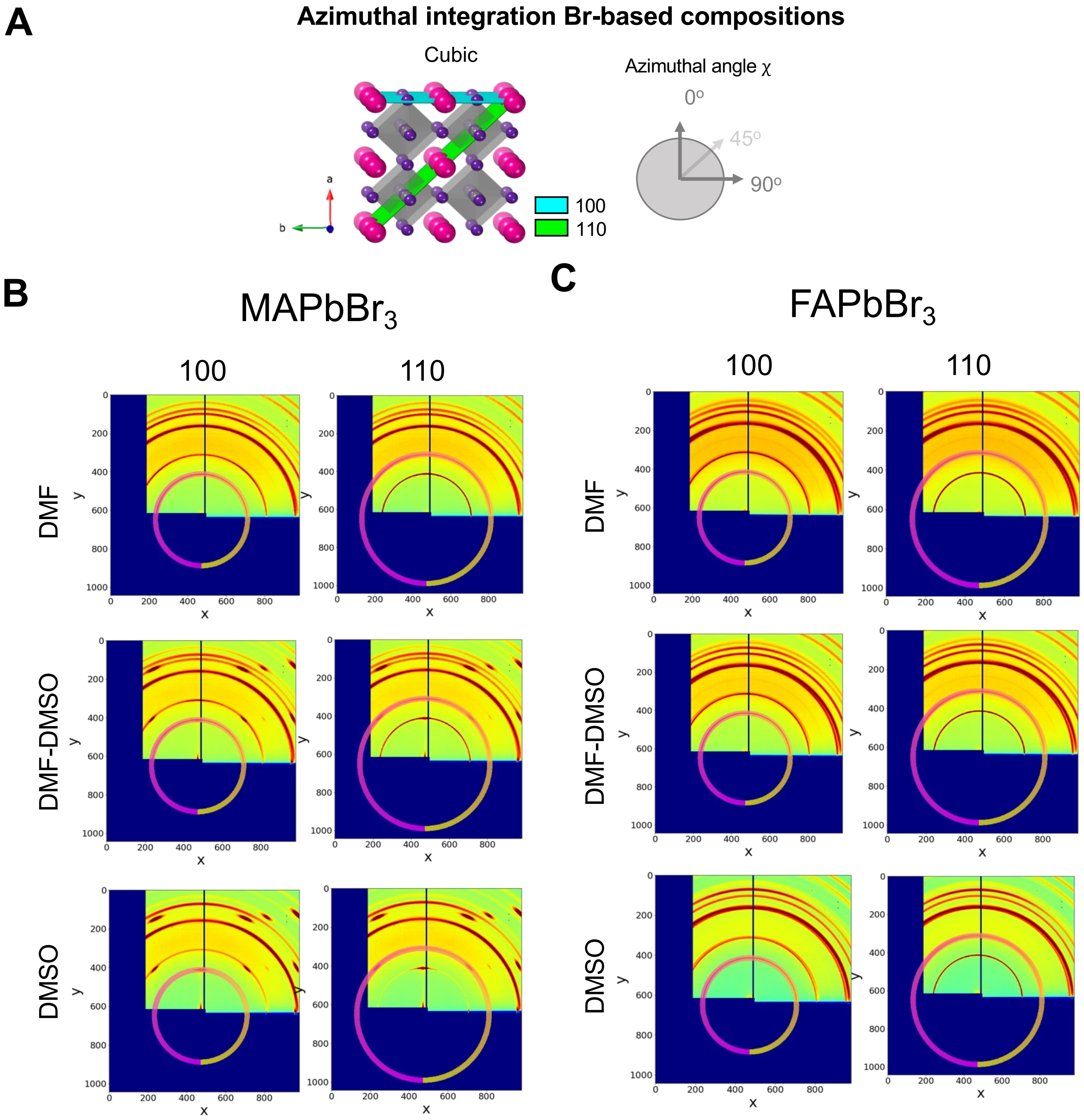

Figure S3. GIWAXS azimuthal ring that is integrated into the azimuthal profiles in Figure 2 of the main text. **(A)** shows the crystal structure, planes, and reference on the azimuthal profile angles. The azimuthal integration is done to the 100 and 110 Debye-Scherrer rings for **(B)** $MAPbBr_3$ and **(C)** $FAPbBr_3$.

Table S1. Azimuthal integration peak fitting parameters

	100 azimuthal peak			
	MA			
	FWHM	error	Height	R2 fit
DMF	21.80	0.223	3473	0.974
DMF:DMSO 4:1	4.61	0.043	9353	0.989
DMSO	4.60	0.015	11032	0.998
	FA			
DMF	22.54	4.550	170	0.004
DMF:DMSO 4:1	29.16	0.780	831	0.777
DMSO	25.65	0.830	687	0.863

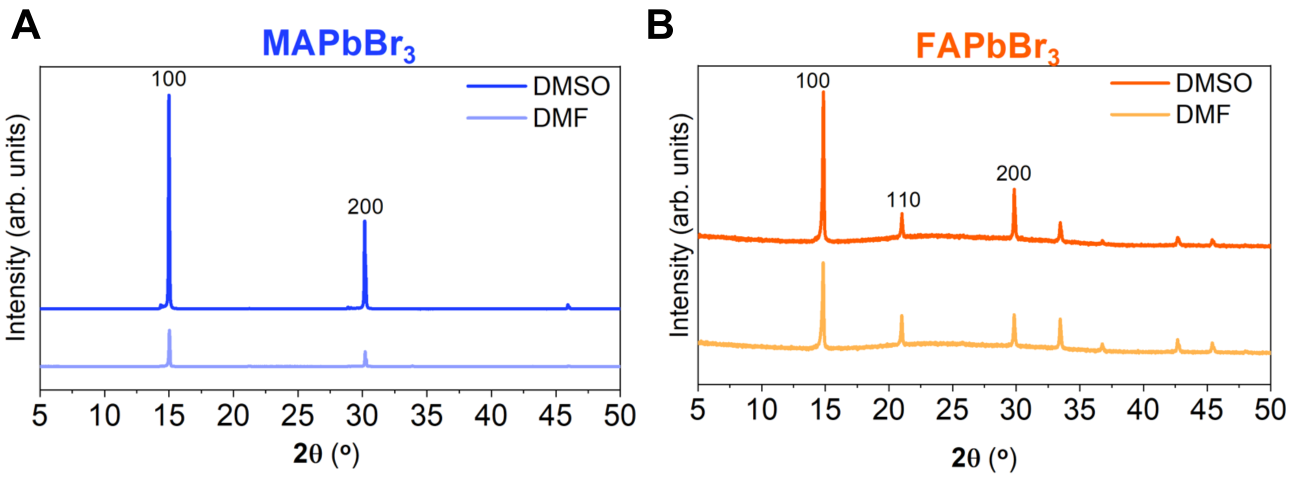

Figure S4. XRD of the deposited thin films in glass, in DMF and DMSO for **(A)** $MAPbBr_3$ **(B)** $FAPbBr_3$

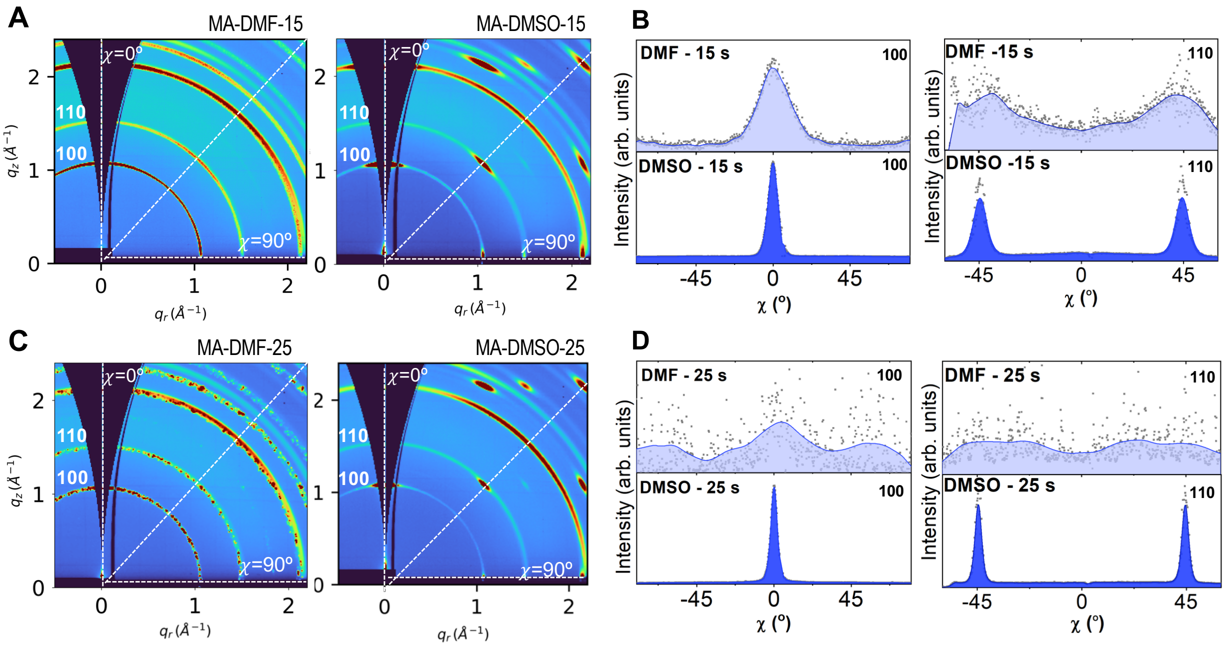

Figure S5. $MAPbBr_3$ perovskite by adding chlorobenzene (CB) at different times in the spin-coating process. **(A)** 15 s for DMF and DMSO, **(B)** Azimuthal integration profiles of the main Debye-Scherrer rings (100) and (110) as a function of χ angle from GIWAXS for DMF or DMSO. **(C)** 25 s form DMF and DMSO, **(D)** Azimuthal integration profiles of the main Debye-Scherrer rings (100) and (110) as a function of χ angle from GIWAXS for DMF or DMSO.

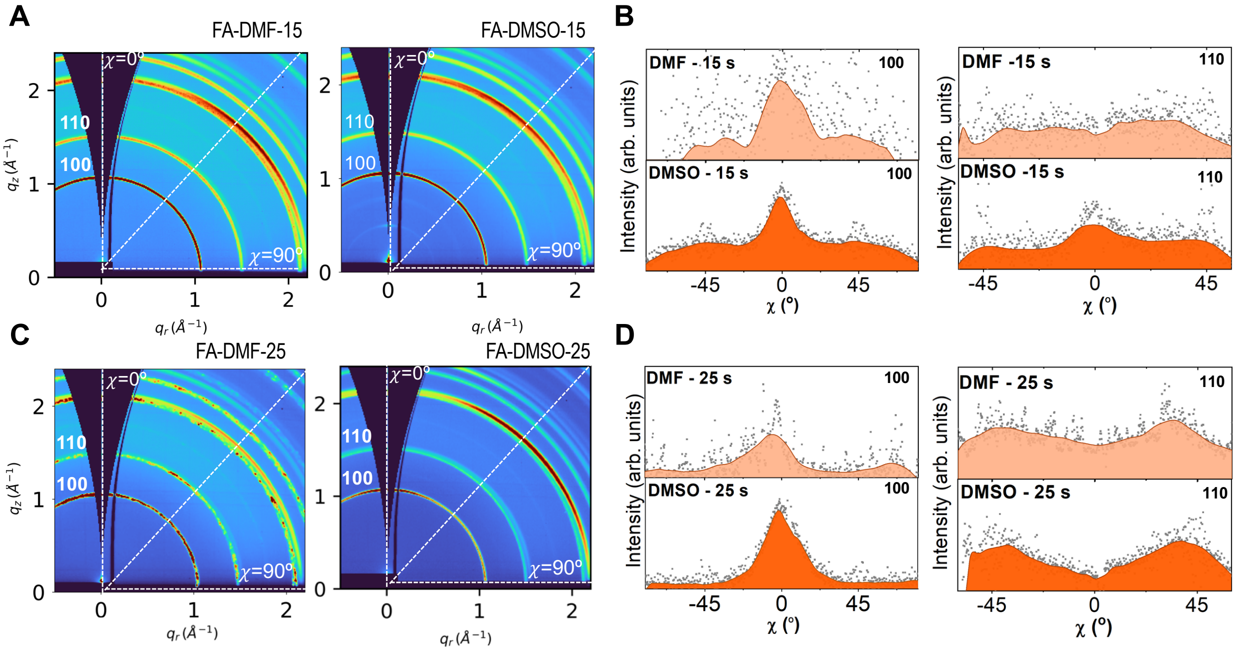

Figure S6. $FAPbBr_3$ perovskite by adding chlorobenzene (CB) at different times in the spin-coating process. **(A)** 15 s for DMF and DMSO, **(B)** Azimuthal integration profiles of the main Debye-Scherrer rings (100) and (110) as a function of χ angle from GIWAXS for DMF or DMSO. **(C)** 25 s form DMF and DMSO, **(D)** Azimuthal integration profiles of the main Debye-Scherrer rings (100) and (110) as a function of χ angle from GIWAXS for DMF or DMSO.

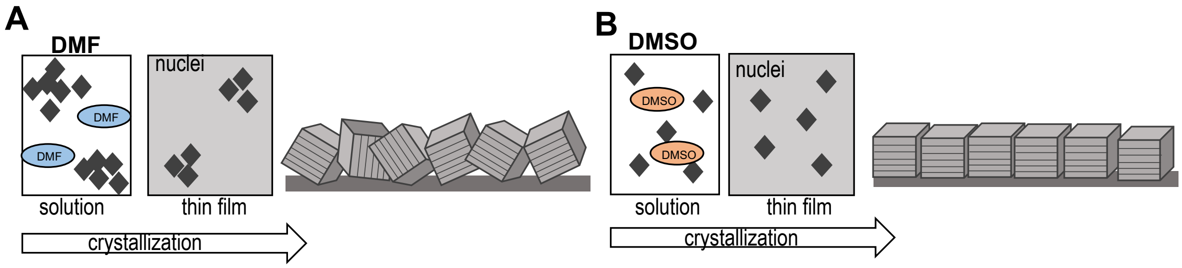

Figure S7. Schematics on the crystallographic orientation by different solvents. **(A)** DMF leads to random orientation, while **(B)** DMSO leads to a high degree of orientation.

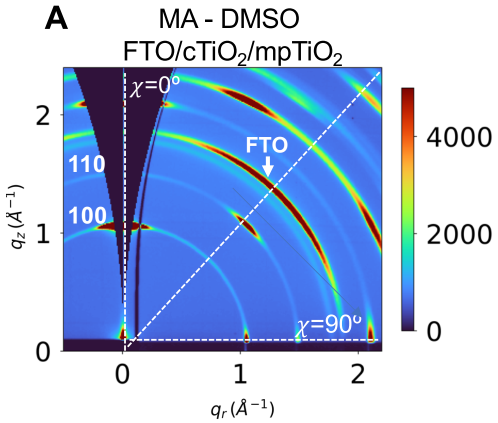

Figure S8. Substrate effect on crystallographic orientation, films deposited on FTO/ compact TiO_2 ($cTiO_2$)/ mesoporous TiO_2 ($mpTiO_2$) layer for **(A)** $MAPbBr_3$ in DMSO, **(B)** $FAPbBr_3$ in DMSO.

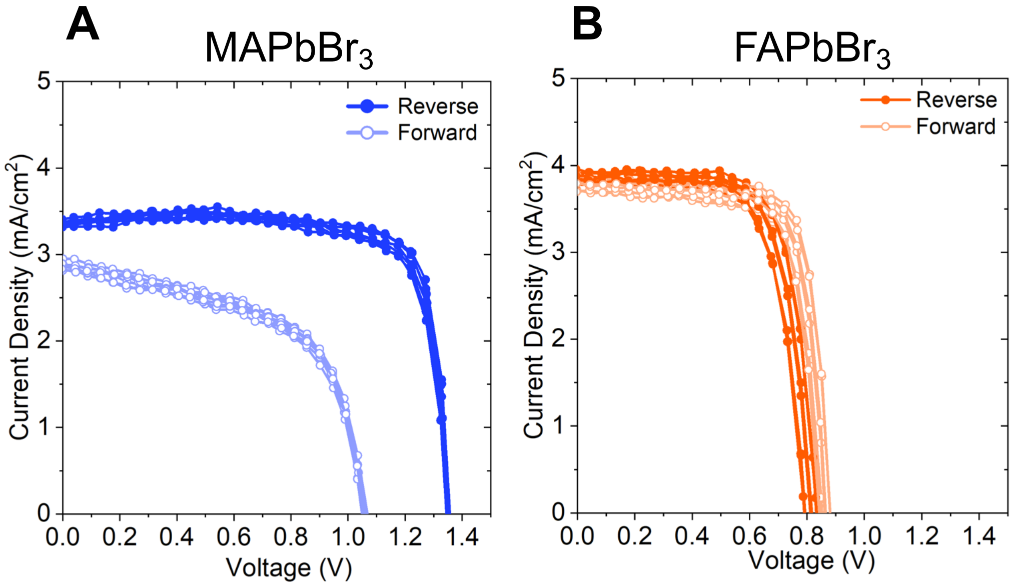

Figure S9. Representative current density – voltage curves for 7 devices of **(A)** $MAPbBr_3$ and **(B)** $FAPbBr_3$

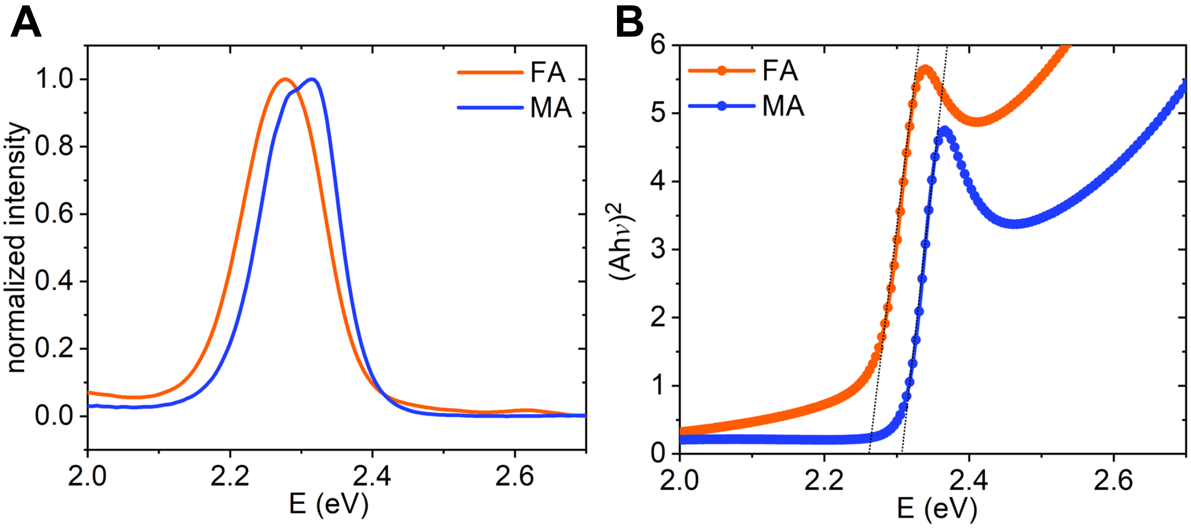

Figure S10. Optical properties of the lead bromide thin films deposited on glass, MA is $MAPbBr_3$ and FA is $FAPbBr_3$. **(A)** Photoluminescence spectra, and **(B)** Absorption spectra from UV-VIS spectroscopy.

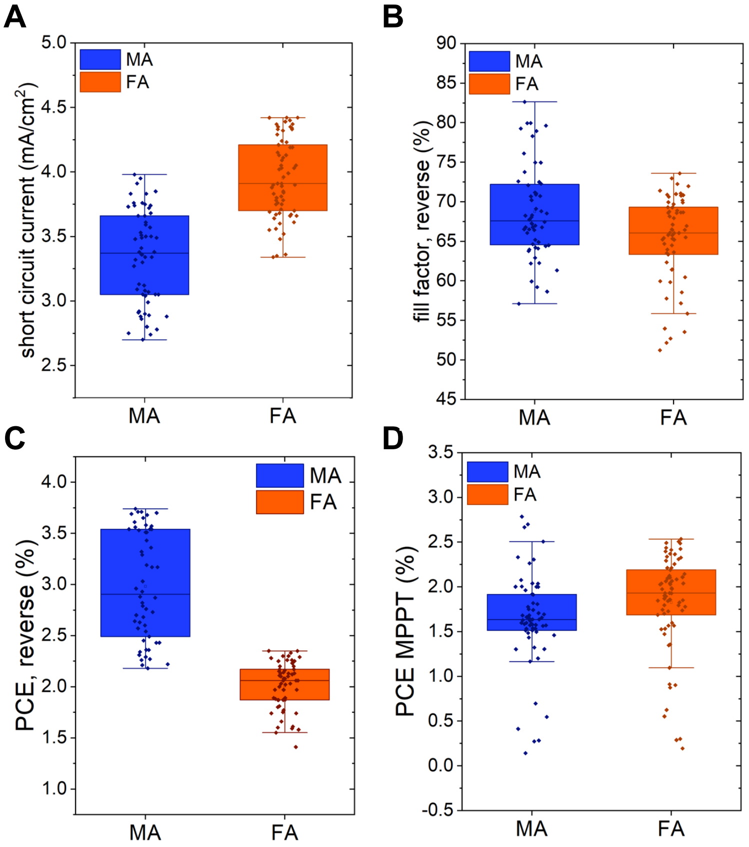

Figure S11. Lead bromide perovskite solar cell parameters MA: $MAPbBr_3$ and FA: $FAPbBr_3$. **(A)** Short circuit current, from reverse scan. **(B)** Fill factor, from reverse scan. **C)** Power conversion efficiency, from reverse scan, **(D)** stabilized power conversion efficiency from the maximum power point tracking.

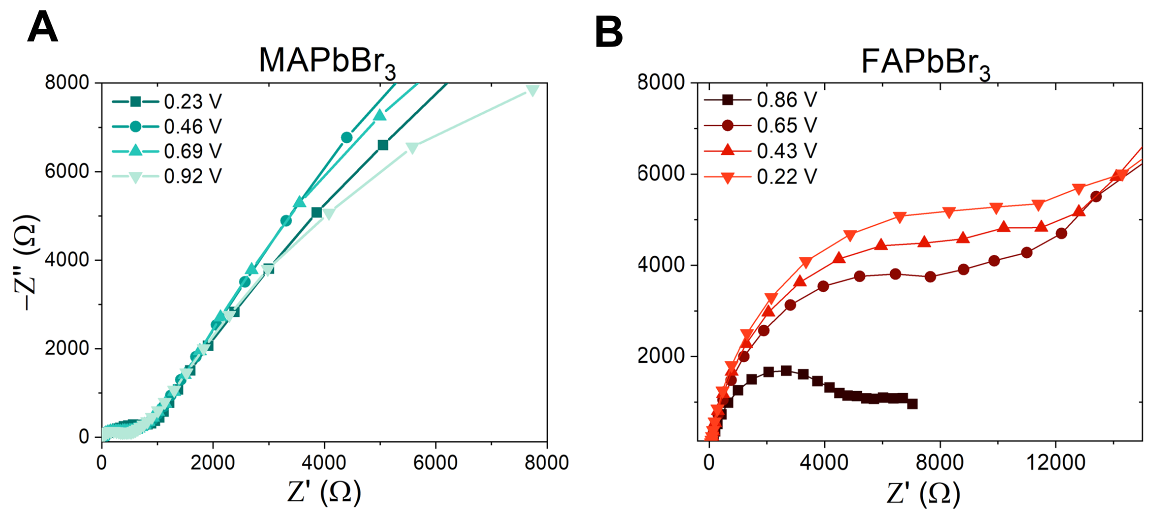

Figure S12. Impedance Nyquist plots for the studied lead bromide perovskites **(A)** $MAPbBr_3$ and **(B)** $FAPbBr_3$.
